# Reducing office workers’ sitting time: rationale and study design for the *Stand Up Victoria* cluster randomized trial

**DOI:** 10.1186/1471-2458-13-1057

**Published:** 2013-11-09

**Authors:** David W Dunstan, Glen Wiesner, Elizabeth G Eakin, Maike Neuhaus, Neville Owen, Anthony D LaMontagne, Marj Moodie, Elisabeth AH Winkler, Brianna S Fjeldsoe, Sheleigh Lawler, Genevieve N Healy

**Affiliations:** 1 Baker IDI Heart and Diabetes Institute, Melbourne, Australia; 2 The University of Queensland, School of Population Health, Brisbane, Australia; 3 School of Sport Science, Exercise & Health, University of Western Australia, Perth, Australia; 4 School of Exercise and Nutrition Sciences, Deakin University, Melbourne, Australia; 5 School of Public Health & Preventive Medicine, Monash University, Melbourne, Australia; 6 The McCaughey VicHealth Centre for Community Wellbeing, Melbourne School of Population and Global Health, The University of Melbourne, Melbourne, Australia; 7 Deakin Health Economics, Faculty of Health, Deakin University, Melbourne, Australia; 8 Curtin University, School of Physiotherapy, Faculty of Health Sciences, Perth, Western Australia, Australia

**Keywords:** Sedentary behaviour, Workplace, Randomised intervention, Office workers, Cardio-metabolic biomarkers, Activity permissive desks, Accelerometry, Physical activity

## Abstract

**Background:**

Excessive time spent in sedentary behaviours (sitting or lying with low energy expenditure) is associated with an increased risk for type 2 diabetes, cardiovascular disease and some cancers. Desk-based office workers typically accumulate high amounts of daily sitting time, often in prolonged unbroken bouts. The *Stand Up Victoria* study aims to determine whether a 3-month multi-component intervention in the office setting reduces workplace sitting, particularly prolonged, unbroken sitting time, and results in improvements in cardio-metabolic biomarkers and work-related outcomes, compared to usual practice.

**Methods/Design:**

A two-arm cluster-randomized controlled trial (RCT), with worksites as the unit of randomization, will be conducted in 16 worksites located in Victoria, Australia. Work units from one organisation (Department of Human Services, Australian Government) will be allocated to either the multi-component intervention (organisational, environmental [height-adjustable workstations], and individual behavioural strategies) or to a usual practice control group. The recruitment target is 160 participants (office-based workers aged 18–65 years and working at least 0.6 full time equivalent) per arm. At each assessment (0- [baseline], 3- [post intervention], and 12-months [follow-up]), objective measurement via the activPAL3 activity monitor will be used to assess workplace: sitting time (primary outcome); prolonged sitting time (sitting time accrued in bouts of ≥30 minutes); standing time; sit-to-stand transitions; and, moving time. Additional outcomes assessed will include: non-workplace activity; cardio-metabolic biomarkers and health indicators (including fasting glucose, lipids and insulin; anthropometric measures; blood pressure; and, musculoskeletal symptoms); and, work-related outcomes (presenteeism, absenteeism, productivity, work performance). Incremental cost-effectiveness and identification of both workplace and individual-level mediators and moderators of change will also be evaluated.

**Discussion:**

*Stand Up Victoria* will be the first cluster-RCT to evaluate the effectiveness of a multi-component intervention aimed at reducing prolonged workplace sitting in office workers. Strengths include the objective measurement of activity and assessment of the intervention on markers of cardio-metabolic health. Health- and work-related benefits, as well as the cost-effectiveness of the intervention, will help to inform future occupational practice.

**Trial registration:**

ACTRN1211000742976

## Background

Current public health guidelines recommend engaging in at least 30 minutes of moderate- to vigorous-intensity physical activity (MVPA) on most days, in order to prevent chronic diseases such as type 2 diabetes and cardiovascular disease [1,2]. However, most adults spend much of their time in environments that not only limit their physical activity, but also require them to sit for prolonged periods of time. Increasingly, evidence suggests that sedentary behaviour may adversely affect health independent of leisure-time MVPA participation [3,4]. Over the past decade, observational studies have demonstrated that total time spent sedentary, and the manner in which sedentary time is accumulated, is detrimentally associated with several health outcomes including elevated markers of cardio-metabolic risk, type 2 diabetes, obesity, cardiovascular disease, breast and colon cancer and premature mortality [5,6]. These adverse health relationships, coupled with the high proportion of the waking day spent in this behaviour [7], have prompted calls for interventions to specifically target a reduction in sitting time [8,9], with a particular focus on high-risk settings such as the office workplace [10-12].

The potential hazards of prolonged sitting in the workplace were first highlighted as early as the 17^th^ century when the distinguished occupational physician Ramazzini [13] reported that relationships between sedentary behaviour and deleterious health consequences were evident in workers whose occupations required them to sit for long hours. In the 1950s, Morris and colleagues [14] reported that workers in occupations requiring primarily sitting (London bus drivers and mail sorters) had a higher incidence of coronary heart disease than did workers who were required to stand and ambulate (bus conductors and postal delivery workers). In recent decades, significant alterations in workplace environments and work practices have occurred, largely driven by technological innovations, such as computers and other labour-saving devices [15]. This was recently highlighted in an analysis of the trends in occupational physical activity during the past 50 years in the USA, showing that there has been a progressive shift away from occupations that require moderate-intensity physical activity to occupations that largely require sitting [16].

Recent observational studies using objective measures of physical activity and sedentary time have provided new evidence on the extent of time spent sitting in the modern workplace – particularly among office-based workers [11,17-20]. In an Australian sample of 193 employees working in offices, call centres and customer service employees, Thorp *et al.* reported that sedentary time (derived from hip-worn accelerometers) comprised more than three-quarters (77%) of total work hours [11], a finding consistent with those observed in Scottish [19] and Swedish workers [21]. In those studies that have specifically assessed the manner in which the sedentary time was accumulated, a considerable amount of workplace sedentary time (22-52%) was accrued in prolonged unbroken bouts (≥ 30 minutes) [19,20,22]. Such extended periods of uninterrupted sitting may have important health implications [23]. Current recommendations, based largely on expert consensus and emanating from musculoskeletal medicine [19,24-26], suggest postural transitions at least every 20–30 minutes. Furthermore, recent experimental evidence has suggested that restricting sitting time durations to those advocated in these recommendations may also provide metabolic benefits, as demonstrated by the acute lowering of post-prandial glucose and insulin with sitting interrupted every 20 minutes with brief (two minute) bouts of activity compared to uninterrupted sitting [27].

Historically, interventions that have specifically addressed workplace sitting have emanated primarily from an ergonomics perspective, with an emphasis on reducing musculoskeletal complaints, rather than reducing sitting time per se [12,28,29]. The associated intervention strategies have included: increasing the number of breaks from sitting time [30-33]; promoting regular postural changes [29,34]; and, ergonomic changes to the individual workspace, including the use of sit-stand workstations [35-37]. Collectively, these studies have demonstrated that frequent changes in posture can have a beneficial impact on musculoskeletal health, either a beneficial or neutral effect on productivity, and were rated as preferable compared to either just sitting or standing [12].

More recently, workplace interventions with a specific public health focus have been developed. Here, the largest reductions in workplace sitting time in the studies to date have been observed when the intervention targeted an individual-level environmental modification (e.g. a sit-stand workstation) [17,38-40]. Individual-based counselling [18] and computer prompt software [22] have also been modestly effective. Small improvements in blood lipids, reduced upper back and neck pain and improved mood states following intervention have been noted [17,39]. However, the methodological limitations of these studies - use of non-randomised study designs, small sample sizes, short follow-up periods and/or poor control for confounding - have precluded definitive conclusions on the possible impact of such interventions on health outcomes or health risk indicators. Larger cluster-randomised trials in non-university staff samples are urgently needed as these: can better control for confounding; improve generalizability; have the capacity to explore effect modification; and, provide more precise estimates of potential effects on anthropometric, biomarker, health, and work-related outcomes. The present *Stand Up Victoria* study has been designed to address these significant gaps in evidence.

### Rationale for the *Stand Up Victoria* intervention

Modern workplace health intervention frameworks draw principally from two disciplines: occupational health & safety (OH&S) and workplace health promotion. OH&S prioritises intervention at the source of the hazard (primary prevention), followed by control of the hazard at the level of the worker (secondary prevention, such as through the use of personal protective equipment), and finally the management of work-related illness or injury if it occurs (tertiary intervention, including treatment, rehabilitation and return to work) [41,42]. Workplace health promotion has traditionally focussed on personal health behaviours that influence chronic disease risk, such as smoking and leisure-time physical activity. More recently, it has been acknowledged that working conditions can also contribute to chronic disease risk and should therefore be targeted alongside health behaviours [41,43,44]. There has been a recent convergence of these two perspectives, as exemplified in the 2010 WHO 'healthy workplace’ model [45]. This framework emphasises that best practice intervention should involve an integrated approach, involving individual, environmental, and organisational-level change components [43,45,46].

The *Stand Up Victoria* intervention follows these principles of an integrated approach: a method that is consistent with ecological frameworks for sedentary behaviour that emphasise the need to consider multiple levels of influence on the behaviour [47].

Here we provide a detailed overview of the *Stand Up Victoria* study including its aims, intervention methods, and evaluation protocol.

## Methods/Design

### Aims

The primary aim of the *Stand Up Victoria* study is to determine whether a 3-month multi-component workplace intervention, incorporating organisational-, environmental-, and individual-level strategies, results in reductions in workplace sitting time (primary outcome) in office workers. Secondary aims are to: determine the impact of the intervention on other activity outcomes (prolonged sitting, standing and moving at work; sitting, standing and moving across the whole day) and on health- and work-related outcomes; identify the factors that mediate and moderate intervention impacts; assess intervention cost-effectiveness; and, evaluate the extent to which changes are maintained 9-months post intervention.

### Study design

**Figure 1:**
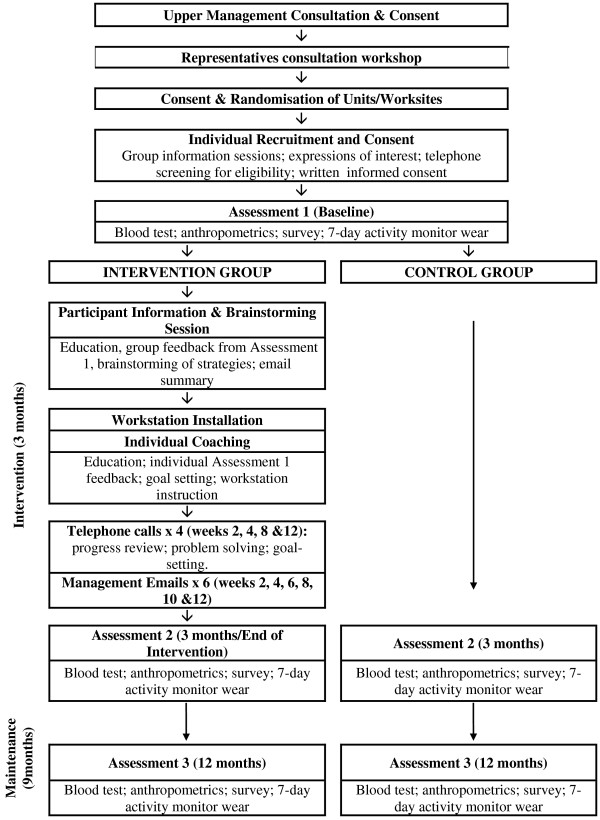
Study overview.

*Stand Up Victoria* is a two-arm cluster-randomised controlled trial in office workers with worksites being the unit of randomisation. The 12-month study protocol includes three assessment time-points: baseline, 3-months (end of intervention) and 12-months (maintenance). A study overview showing the major components and time-points is given in Figure 1. *Stand Up Victoria* is funded by a National Health and Medical Research Council (NHMRC) Project Grant (#1002706) and the Victorian Health Promotion Foundation (VicHealth). Ethics approval was granted by Alfred Health Human Ethics Committee (Melbourne, Australia). Contract and tender approval with the partner organisation was provided by senior management. The study will be conducted in accordance with the CONSORT guidelines (http://www.consort-statement.org/).

### Study population

Participants will be office workers, recruited from 16 different study sites from the one organisation. The aim is to recruit 160 participants per condition (see sample size calculations). Participant eligibility will be based on working at least 0.6 full time equivalent hours (FTE), aged 18–65 years, speaking English, and having designated access to a telephone, internet and desk within the workplace. Participants will be excluded from the study if they are pregnant, non-ambulatory, have a pre-existing musculoskeletal complaint, and/or have a planned absence from work for >2 weeks or a planned relocation to another workplace during the 3-month intervention period.

### Recruitment

#### *Recruitment of organisation*

A multi-site organisation has been recruited via a tender process to be a partner in this project through provision of its’ workplaces and employees as participants. The selected organisation, Department of Human Services (DHS), is the Australian government department responsible for the delivery of social and health-related payments and services including employment benefits, student benefits, child support payments and Medicare benefits. In 2011–2012, DHS employed 36, 977 staff across 523 service centres Australia-wide (http://www.humanservices.gov.au).

#### *Recruitment of study sites*

DHS workplaces (i.e. study sites; defined as geographically separate DHS buildings) within the state of Victoria that are not currently delivering a physical activity program for their staff will be eligible to participate. Sites will be identified by a DHS employee designated as the research liaison person. Within each site, a team (i.e. a distinct working group within the site that has a line manager and regular group meetings and interactions) will be identified. If the team size falls below a threshold of <10 employees, a second (small: <10 employees) team from the same location will additionally be invited to participate. Written consent will be obtained from the appropriate divisional manager(s) of each team participating at eligible sites. Information sessions will be conducted by the research team for the appropriate managers outlining the study requirements and expectations from both individual employees and the team. In addition, appropriate management support will be sought for the environmental component to be incorporated into the office workspace and for health coaching elements to be conducted during work time. Managers will be asked to provide written informed consent and express unreserved commitment to having their employees participate in the study. Consenting sites will be randomised to either the intervention or control arms of the trial.

#### *Recruitment of participants*

An initial information session (20–30 minutes) will be delivered by research staff to employees of consenting sites. At this session, employees will be given detailed information on the study and the required commitment. Following this session, an expression-of-interest form will be distributed for return either to their worksite team leader or directly to the research staff. Employees interested in participating will be subsequently assessed for eligibility via a telephone-administered interview. During the telephone interview, the research coordinator will explain the study, assess eligibility, and solicit verbal willingness to participate. If the employee is eligible and interested, they will then be emailed the participant information and consent form. Participants will be considered formally enrolled upon return of the signed consent form prior to commencement of study assessments.

### Randomisation

Randomisation to either the intervention or control arms of the trial will be at the level of the study site. Simple cluster randomisation will be achieved by generating a randomisation plan for up to 24 clusters/worksites in one block (http://www.randomization.com). Participating sites will subsequently be randomly matched against the randomisation plan using a list randomiser (http://www.random.org). This method allows study staff to implement the study intervention and control protocols in an order consistent with logistical capacity. Study sites will be enrolled and then randomized until the required number of clusters and sample size is reached.

### Control – usual practice

Participants within the control sites will be advised that the aims of the study are to examine the consistency of patterns of physical activity and sedentary time in office workers, and how these may be associated with cardio-metabolic and anthropometric markers. The control group will receive the same assessments at the three time points as the intervention group.

### Intervention

#### *Theoretical basis & intervention development*

*Stand Up Victoria* is a multicomponent intervention comprising organisational, environmental and individual elements. Extensive formative research was used to guide intervention development and is described in a separate paper (Manuscript Submitted). In brief, this included testing of each intervention element on small samples of office workers, including an evaluation of the effectiveness and acceptability of the sit-stand workstations [17]. The formative research culminated with a two-group (intervention versus control) pilot study (*Stand Up Comcare*) in which all intervention elements were combined and evaluated in an abbreviated version of the intervention [20]. The intervention is based on Social Cognitive Theory, with emphasis on the constructs of self-efficacy, outcome expectancies and socio-structural factors [48]. An intervention taxonomy of behaviour change strategies [49] guided the translation of the theoretical components into intervention strategies (described below). The intervention will be delivered over three months, with this duration being consistent with the median length of interventions included in a recent meta-analysis of workplace physical activity intervention trials [50].

#### *Intervention messages*

The intervention comprises three key strategic messages: “Stand Up, Sit Less, Move More”. 'Stand Up’ is a prompt to break-up prolonged bouts of sitting. The aim is to reduce musculoskeletal symptoms and to promote beneficial physiologic changes associated with regular, frequent muscle activation [27,51]. Building on both OHS guidelines [25,41,42], as well as recent experimental evidence [27,51], the recommendation is to interrupt sitting at least every 30 minutes with postural change. 'Sit Less’ communicates a reduction in overall sitting time by substituting some sitting with either standing or moving, aiming for an approximately equal sit to stand ratio over the course of the day. Here, the workstation is promoted as a primary means for reducing overall sitting time. Finally, the principle of 'Move More’ is to increase physical activity throughout the working day; primarily through opportunistic, incidental activity.

#### *Intervention procedures*

##### *Organisational elements*

The organisational level of the intervention will include three key elements: a senior management consultation; a representatives’ consultation workshop; and, a participant information and brainstorming session (see Figure 1). Visible ongoing organisational support will be demonstrated through the distribution of tailored emails from management.

#### *Senior management consultation*

In order to establish the study, senior research staff met with senior DHS management to describe the background and rationale of the study and the study timeline, and obtain consent for the trial. This involved obtaining approval for intervention units to have physical work environment modifications (detailed below) as well as other intervention activities at the organisational and individual levels. Current organisational processes and structures important to study implementation were considered, including potentially eligible worksites. Strategies to encourage employee participation were discussed and relevant OHS policies and resources identified (e.g. policies relating to workplace activity). The organisation has dedicated a staff member as the research liaison person (at 0.4 FTE for the duration of study; paid by the organisation), aiding in recruitment of work teams into the trial, as well as organising logistics for all assessments and work environment modifications.

#### *Representatives consultation workshop*

Following the senior management consultation, a workshop (approximately 4 hours), facilitated by senior research staff, will be conducted with DHS staff representatives. Representatives will include employees from the respective levels of staff (including general staff and senior and middle managers) as well as other stakeholders including OHS personnel, workplace safety advisors, and corporate ergonomists. During the workshop, the research staff will provide information on the background and rationale of the study, the target behaviour, and the key intervention messages. The representatives will then brainstorm feasible strategies to *Stand Up, Sit Less, Move More* within DHS. Team champions will be identified, and their role described (further details below).

#### *Participant information and brainstorming session*

Following baseline assessment, an information and brainstorming session (approximately 30–45 minutes) will be delivered by research staff to participants at each study worksite. Each session will: (1) outline recent research findings on the health consequences of excessive sitting using standard materials developed by the research team; (2) provide summary feedback of device-measured sitting and activity time generated from the group’s baseline assessment; and, (3) include a brainstorming component. Strategies identified in the representatives’ consultation workshop will be discussed, with further brainstorming from all staff to identify and agree upon strategies that are specifically suitable for their local worksite. As part of this brainstorming session, the Heart Foundation of Australia consumer information sheet “Sitting less for adults” containing tips on how to reduce sitting time in the workplace [52] will be used to facilitate discussion and to prompt further ideas. An email summary (sent by the research team) of the information and consultation session will be sent to all participants. This email will include an electronic information booklet (Additional file 1: Figure S1) containing information on the background and rationale for the study; general guidelines on achieving optimal workplace activity; specific behaviour change strategies related to the key intervention messages; and, general information about the study procedures and timeline.

#### *Ongoing organisational support*

Team champions (typically the worksite team leader) will be encouraged to actively promote participation in the study and the implementation of the organisational-level strategies identified in the brainstorming sessions. Team champions will also facilitate communication between participants and research staff and have the responsibility for sending the tailored management emails. The purpose of the management emails is to foster a sense of management support for the key intervention messages. Six email templates, featuring themes of the detrimental effects of prolonged sitting and the benefits of standing and moving more, will be provided to team champions at weeks 2, 4, 6, 8, 10 and 12 for them to send to participants within their team (example provided in Additional file 2: Figure S2). Notably, the second management email (week 4) also includes a list of strategies to reduce sitting time outside the workplace, which parallels the purpose and content of the week 4 telephone support call (see below). Team champions will be encouraged to personalise the templates by highlighting particularly successful strategies to *Stand Up, Sit Less and Move More* within their worksite. The research team will be blind copied on the emails to inform evaluation of intervention fidelity.

### Environmental element

A dual-screen sit-stand workstation (*Ergotron WorkFit-S;*http://www.ergotron.com), including worksurface accessory (a flat surface positioned above the keyboard), will be provided to all intervention participants for the duration of the study (12 months). The workstation allows the participant to easily and quietly alternate their working posture between sitting and standing. It uses minimal desk space, and the keyboard and monitor can move independently to ensure that the appropriate ergonomic posture is maintained. Participants will receive written instructions and tips on the correct ergonomic posture for both sitting and standing, as recommended by the product manufacturer (http://www.ergotron.com/tabid/305/language/en-AU/Default.aspx; Additional file 3: Figure S3). Adhesive stickers will be applied to the workstations by research staff to indicate the recommended configuration – i.e. keyboard and screen position – tailored for each individual for both sitting and standing postures.

### *Individual elements*

Individual-level support for behaviour change delivered to each participant will consist of a face-to-face health coaching session (1–3 days following workstation installation; approximately 30 minutes) and four telephone calls at weeks 2, 4, 8 and 12 (7–10 mins each) following the individual session. Where feasible, the same health coach will remain the point of contact for each participant throughout the study to facilitate rapport. All health coaches will have at least a bachelor’s level training in psychology or a related discipline and will receive training in motivational interviewing techniques adapted for study purposes [53]. Detailed intervention scripts will be used for coach training with accompanying checklists used during intervention delivery to maintain intervention fidelity. Further, health coaches will debrief regularly (monthly for the first few months and then quarterly) via telephone with chief investigators responsible for this element of the intervention (EE, GH) during the active intervention phases.

#### Individual coaching

The coaching will be used to: explain the *Stand Up, Sit Less, Move More* intervention targets; to feedback participant’s assessment results on the extent to which the participant is meeting these targets (derived from the activity monitors worn at baseline; Additional file 4: Figure S4); and, to identify specific goals and behaviour change strategies relating to each of these key intervention messages. The strategies identified in the brainstorming session will be shown to participants who will be encouraged to develop their own strategies as appropriate. A laminated workstation tracker (Additional file 5: Figure S5) will be provided for participants to record their goals and strategies. They will be encouraged to place this within eyesight of their workstation for self-monitoring purposes. Health coaches will record these goals on their own intervention worksheet, which will be referred to during the telephone calls. During this face-to-face session, participants will also receive instruction on the ergonomic setup and appropriate use of the workstation. This will include specific instructions to “listen to their body”, and to regularly change posture (i.e. to neither sit or stand for too long). Following the consultation, a personalised email summary of the session will be sent to participants from the health coach.

#### *Telephone calls*

Four telephone calls will be used to support goal attainment. They will involve assessment of participant progress toward previously set goals, problem-solving as necessary, and adjustment/progression of goals and related behaviour change strategies. The second phone call (week 4) will also address strategies to reduce sitting time outside the workplace. Health coaches will record notes about individual’s progress and amended goals, while participants will be encouraged to update their workstation tracker accordingly. Call attempts, completions, and duration will be tracked, and a call content checklist completed after each call to inform an evaluation of intervention fidelity.

### Data collection

Assessments will be undertaken at baseline, 3-, and 12- months in designated testing rooms at the respective worksites by trained project staff. Participants will receive verbal and written requests (with confirmation of compliance at data collection time) to refrain from any MVPA, alcohol, and caffeine in the 24 hours preceding each assessment, and to fast for at least eight hours. This is to minimise any potential confounding with respect to biomarker outcomes. A fasting blood sample will be collected on-site and the sample analysed by an accredited pathology laboratory. Sitting, standing, and moving time outcomes will be collected via activity monitors in the seven days following the onsite assessment. Participants will receive full instruction on the use of the activity monitors and the accompanying daily log. The devices and the daily logs will be collected by research staff at the end of each seven day period. A self-administered questionnaire will be completed online using the LimeService (http://www.limeservice.com) survey creation and hosting platform. Participants will access the survey using unique tokens contained within an email invitation. This ensures both exclusivity and confidentiality. In addition, descriptive information relating to the respective intervention worksites (e.g.,: office layout and stair availability will be obtained by research staff. Full details of the measures are provided below and in Table 1.

**Table 1:** Summary of measures used in the Stand Up Victoria office workplace intervention

**Behavioural**	**Objectively measured physical activity and sitting time**
*Objective & self-report*	*activPAL3*
	**● Workplace sitting time (primary outcome)**
	**● **Prolonged (>= 30 mins) workplace sitting time
	**● **Workplace standing time
	**● **Workplace moving time (stepping time, no. steps)
	**● **Number of sit-stand transitions at workplace
	**● **Non-workplace sitting, standing, moving time
	*Actigraph GT3X+*
	**● **Average daily MVPA
	**● **Average daily light physical activity
	*Self-report measures*
	**● **% sitting, standing, walking and physically demanding tasks at work
	**● **Recent work attendance (days/week; hours/week)
	**● **Non-workplace sitting time; TV/video viewing time
	**● **Diet (fat intake; fibre intake)
	**● **Smoking status
**Anthropometric***Objectively measured*	**● **Height (baseline only)
	**● **Weight
	**● **BMI (kg/m^2^)
	**● **Waist circumference
	**● **Hip circumference
	**● **Waist-Hip ratio
	**● **Body composition (% and kg fat and fat-free mass)
**Cardio-metabolic***Objectively measured*	**● **Fasting blood glucose
	**● **Fasting insulin
	**● **Cholesterol (total, HDL, LDL)
	**● **Triglycerides
	**● **Blood pressure
**Socio-demographic***Self-report*	All baseline only
	**● **Age
	**● **Gender
	**● **Race/ethnicity
	**● **Marital status
	**● **Education
**Health status** Self-report	**● **History of diabetes & hyperlipidaemia (baseline only)

	**● **Musculoskeletal health
	**● **Eye strain
	**● **Stress-related symptoms (fatigue, headaches, digestive problems, sleep quality)
**Work** Self-report, internal DHS measure, and employee records	**● **Employment status (baseline only) including length of tenure; job classification; full-time equivalent (FTE) level
	**● **Secondary employment status (including FTE)
	**● **Productivity
	**● **Presenteeism/absenteeism
	**● **Work performance
**Psychosocial-Environmental**Self-report and objectively measured	**● **Perceptions of the work environment
	**● **Desk/workstation utilisation
	**● **Frequency and duration of working with colleagues as well as perceived adequacy of space(s) for such interactions.
	**● **Quality of life
	**● **Acceptability of workstations (intervention group only)
	**● **Preference for sitting and standing in the workplace
	**● **Knowledge
	**● **Barrier self efficacy
	Perceived behavioural control
	**● **Perceived organisational social norms
	**● **Use of self-regulation strategies
	**● **Use of intervention-specific strategies
	**● **Descriptive office audit
**Cost-effectiveness**Self-report	**● **Health-related quality of life
	**● **Health care utilisation
	**● **Cost to deliver intervention
	**● **Adverse events (intervention group only; 3 and 12 months only)

All measurements completed at baseline, 3- and 12-months except where noted.

### Outcomes

#### *Sitting, standing and moving time*

Sitting, standing, and moving time will be objectively measured via an activPAL3 activity monitor (PAL Technologies Limited, Glasgow, UK; default settings). This monitor continuously records the precise beginning and ending of each bout of sitting or lying (here termed sitting), standing, and stepping at a variety of speeds, and the estimated MET-hours expended during those bouts. Previous studies have shown this device to be valid, reliable and responsive [54-57]. Waterproofing of the device will be achieved by first inserting it into a nitrile finger cote and then wrapping the device in waterproof *Opsite Flexifix*^*(™)*^ (Smith & Nephew).Thereafter, the activPAL3 will be secured to the anterior mid-line of the right thigh, about a third of the way down from the hip, using hypoallergenic adhesive material (*Hypafix®,* BSN medical). Additional hypoallergenic patches will be given to participants for the adhesive materials to be changed as required. Participants will be requested to wear the activPAL3 activity monitor for 24 hours per day, for seven consecutive days at each assessment period (baseline, 3-, and 12-months). At each assessment, participants will also concurrently wear the tri-axial GT3X + Actigraph activity monitor (ActiGraph, Pensacola, Florida). Participants will be asked to wear this activity monitor during waking hours only (except for water-based activities) for the seven-day assessment period. The accelerometer is positioned over the right hip via an elastic belt. The raw accelerometer data will be collected at 30Hz.

Daily logs (self-completed) will be used to record wake and sleep times, work hours (defined as time spent at the primary DHS study worksite), and any device removal greater than 15 minutes. Periods of work time spent not at the primary worksite (i.e. working from home) will also be recorded. A customized SAS 9.3 (SAS Institute Inc., Cary, NC) program, utilising both the activity monitor and log data, will be used to generate sitting, standing, and moving outcomes at work and overall, with the primary outcome being sitting time at work (measured by activPAL3). Consistent with the intervention message, prolonged sitting is defined as time accrued in sitting bouts at least 30 minutes in length. The number of transitions between sitting and standing will also be measured. The GT3X + activity monitor will be used to differentiate time spent in light-intensity physical activity and MVPA.

#### *Anthropometry: height, weight and body composition*

Waist circumference will be measured (nearest 0.1 cm) with a non-expandable tape at the midpoint between the lowest rib and the iliac crest. Hip circumference (nearest 0.1 cm) will be taken as the maximum circumference in the horizontal plane, measured over the buttocks. For both waist and hip measures, measurements will be taken in duplicate with a third measurement taken if the first two differ by ≥ 1 cm. Fat mass, fat-free mass and percent body fat will be measured using foot-to-foot bioelectrical impedance analysis (BIA) scales (Model TISC-330S, Tanita Inc., Tokyo, Japan) in the fasted and voided state. Bioimpedance analysis will not be conducted on participants with a pacemaker. Weight will concurrently be measured using the same BIA scales without shoes and wearing light garments to the nearest 0.1 kg. Standing height will be measured to the nearest 0.1 cm, without shoes and the individual’s eyes looking straight ahead (Frankfort plane), using a portable stadiometer. Height will be measured in duplicate with a third measurement taken if the difference is ≥ 0.5 cm. Body mass-index (BMI; kg/m^2^) will be calculated using the average height and weight obtained from the above measures.

#### *Cardio-metabolic markers*

Fasting blood samples will be collected on-site in the morning by a trained phlebotomist for the analyses of glucose, lipids (triglycerides, high density lipoprotein [HDL] cholesterol and low density lipoprotein [LDL] cholesterol) and insulin. Samples will be sent immediately to an accredited testing laboratory for analysis (Melbourne Pathology). Fasting plasma glucose will be measured by spectrophotometric-hexokinase method. Fasting total cholesterol, HDL- cholesterol and triglycerides will be measured via standard enzymatic-colorimetric methods. LDL-cholesterol will be estimated using the Friedewald equation [51]. Insulin will be measured by electrochemiluminescence immunoassay (ECLIA). All blood chemistry analytes will be measured using Roche/Hitachi cobas® system analysers (Tokyo, Japan). Blood pressure will be measured via a digital blood pressure monitor (OMRON HEM-907; Omron Healthcare, Japan) using the right arm and an appropriately sized cuff. Participants will rest in the seated position for 15 minutes prior to having a minimum of two measurements taken at one-minute intervals. A third measurement will be taken if the systolic differs by >10 mmHg or the diastolic by >6 mmHg.

#### *Survey measures*

**Socio-demographic characteristics** Based on questions used in the Australian Diabetes, Obesity and Lifestyle (AusDiab) study [58], information relating to age, gender, ethnicity, marital status, and education will be obtained (baseline assessment only).

**Physical health history data** Musculoskeletal health will be measured using the 27-item Nordic Musculoskeletal Questionnaire, modified to refer to the last seven days and the last three months (instead of 12 months) [59]. This questionnaire includes items on 'trouble’ in numerous body parts as well as the capacity to perform normal activities in the presence of any 'troubles’, and has been shown to be repeatable and sensitive to change [60]. Eye-strain will be assessed with three items used in a previous ergonomics intervention study, where it was shown to have high internal consistency [61]. A checklist, adapted from previous work that has demonstrated good internal consistency [62], will assess physical health symptoms commonly associated with stress such as fatigue, headaches, digestive problems and sleep quality. Current smoking status (including at work) and history of diabetes and hyperlipidaemia will also be collected.

**Self-reported physical activity and sitting time** Participants will be asked to estimate the total time spent watching TV/videos during the week and on weekends; average daily sitting time during the week and on weekends; and the proportion of sitting, standing, walking and physically demanding tasks during a typical work day in the previous seven days [63,64].

**Work outcomes** Productivity, presenteeism, and absenteeism will be obtained for each assessment period using internal DHS measures and validated questionnaires [65-67]. The Health and Work Questionnaire (HWQ) has six sub-scales (productivity, concentration/focus, supervisor relations, non-work satisfaction, impatience/irritability), with internal consistency scores ranging from alpha = 0.72 to 0.96 [65]. In addition, a total HWQ score will be calculated (alpha = 0.81) [65]. Self-reported work performance will be assessed on a 9-item, 10-point scale [66]. Performance items include amount and quality of work accomplished, meeting deadlines, frequency of errors, taking responsibility, creativity, getting along with others, dependability and overall performance [66]. Presenteeism and absenteeism will be assessed using the proprietary Work Limitations Questionnaire (WLQ), which examines the frequency of difficulty to perform specific job tasks [67].

**Work history and environment** Questions will assess perceptions of the work environment and current work patterns including: length of tenure; job classification; FTE level; desk/workstation utilisation; environmental satisfaction; and, frequency and duration of working with colleagues as well as perceived adequacy of space(s) for such interactions. Previously validated instruments [35,66,68] and items developed specifically for this study (Additional file 6: Figure S6) will be used.

**Dietary intake** will be measured using the 20-item Fat & Fibre Behaviour Index which asked about eating habits over the previous month. This questionnaire has previously been used in our randomised controlled trials and has been shown to be sensitive to change [69].

**Mediators** Potential mediators of change have been conceptualised under the three levels of intervention (organisational, environmental, individual). All mediators will be assessed in both groups at all assessments via the on-line questionnaire. The organisational mediator to be assessed will be the site-specific team champion’s attitudes and knowledge (scales described below). At the environmental level, participants will be asked to report the frequency of use of their workstation in the past month on a 5-point Likert scale (from 'never’ to 'very often’). Individual-level mediators will include the following theoretical constructs: preference for sitting and standing at work; knowledge; barrier self efficacy; perceived behavioural control; perceived organisational social norms; as well as frequency of use of self-regulation strategies and other individual-level intervention strategies. There are no existing measures for these individual-level constructs in relation to workplace sitting; therefore, where possible, we have adapted scales from the more developed physical activity literature or otherwise a study-specific scale was created. All of the scales were pilot tested in our previous workplace sitting intervention [20] and the psychometric properties (internal consistency and test-retest reliability) of these scales can be found in Additional file 7: Table S1. Preference will be measured across two items on a 5-point scale indicating the proportion of work time participants preferred to be sitting or standing (ranging from 'none of the time’ to '80-100% of time’). Knowledge of key intervention messages will be assessed across five items (e.g., “Sitting for most of the time at work is bad for my health”); on a 5-point Likert scale ('strongly disagree to 'strongly agree’). The barrier self efficacy scale has been adapted from an existing scale [70] and will assess nine items referring to specific barriers to reducing workplace sitting (e.g., confidence to 'stand up during meetings at work, even though no one else was’), which will be assessed on a 5-point Likert scale ('not at all confident’ to 'very confident’). Perceived behavioural control will be examined across five items (e.g., 'It is my choice whether I stand up or sit during a meeting with colleagues at work’) on a 5-point Likert scale ('strongly disagree to 'strongly agree’). Organisational social norms will be assessed in eight items (e.g., 'My workplace is committed to supporting staff choices to stand or move more at work’) on a 5-point Likert scale ('strongly disagree to 'strongly agree’). Self-regulation will be examined across 10 items on a 5-point Likert scale ('never’ to 'very often’), adapted from an existing scale for physical activity [71] and will include self-regulation strategies targeted in the intervention (e.g., “recorded my sitting or standing at work in a written record”). In addition, frequency of use of intervention-specific strategies is also assessed across nine items on a 5-point Likert scale ('never’ to 'very often’). The complete set of questions is provided in Additional file 8: Figure S7.

**Moderators** These will be assessed at baseline in both study groups. Potential moderators will be grouped as: demographic (e.g., age, gender, BMI, health status); work-related characteristics (e.g., position, hours worked per week, main work tasks); office environment characteristics assessed as part of the baseline workplace descriptive audit (e.g., office layout); and behavioural characteristics (e.g., MVPA, sitting outside work hours).

**Adverse events** The adverse events that the participant attributes as “study-related” will be collected at each follow-up assessment in the intervention group only. Health care utilisations (number of visits to GPs and allied health care professionals) pertaining to the adverse event (s) will also be measured as part of the economic evaluation (Additional file 9: Figure S8).

**Quality of life** will be measured using the validated Australian Quality of Life Survey (AQoL-8D) which consists of eight separately scored dimensions (Independent Living, Happiness, Mental Health, Coping, Relationships, Self Worth, Pain, Senses) totalling 35 items [72].

### Qualitative interviews

At the end of the intervention (post 12 months), interviews will be undertaken with a sample of intervention participants, all intervention team leaders, and senior DHS management. The sample of intervention participants (a minimum of two from each intervention worksite) will be randomly selected from those who “opt-in” for this element on the final assessment questionnaire. The interviews will be semi-structured and delivered face-to-face by one research staff member. The interviews with participants and team leaders will include evaluation of the different intervention elements and adherence to the study site strategies identified and agreed upon within the group information session, as well as perceived organisational support for the key messages. The interviews with the senior management will also be semi-structured and delivered face-to-face, with the objectives being to canvass perspectives on the implementation of the intervention and next steps relating to the broader translation/dissemination of similar initiatives within the organisation.

### Economic evaluation

An economic evaluation will be undertaken alongside the trial to determine whether the intervention represents 'value-for-money’ measured against the control group (current practice). It will address issues of both technical efficiency ('how to do it’) through assessment of key design features of the intervention and the associated cost drivers, and allocative efficiency ('what to do’) through the modelling of longer term consequences and cost offsets. In addition to a 'trial-based evaluation’ (costs and outcomes exactly as per the trial), a 'modelled economic evaluation’ will also be undertaken, which extends the target population, time horizon and decision context.

### Feedback of study outcomes

Individual feedback on baseline patterns of sitting, standing and moving time both at the workplace and across all waking hours will be received by intervention participants as part of their one-on-one consultation to facilitate goal setting (Additional file 4: Figure S4). At the completion of both the 3- and 12-month assessments, all participants will receive individual feedback letters containing details of their average sitting, standing, and moving time (both at the workplace and overall) and their anthropometric and cardio-metabolic outcomes, including sex- and gender-specific reference/desirable ranges where applicable. This feedback will also include details of change from the previous assessment(s). Participants will be encouraged to consult their doctor to discuss any cardio-metabolic results outside the desirable range. To ensure participant safety, medical results requiring urgent attention will be communicated to the participant as soon as the health concern is identified.

### Sample size

Minimum differences of interest (MDI) for activity outcomes were 45 minutes of sitting, standing, prolonged sitting, and light activity, and 15 minutes of stepping and MVPA. Based on our study design, we expected an average of 20 participants/cluster, with strong clustering (ICC = 0.1; Design effect = 2.9) for activity (heavily influenced by workplace) and weak clustering (ICC = 0.01; Design Effect = 1.19) for the other outcomes (weakly influenced by workplace) with overall attrition of 30%. Standard deviations and pre-post correlations were assumed based on the earlier pilot [20].

Based on these assumptions, the sample size required to detect the MDI (45 min) for the primary outcome (workplace sitting, assumed SD = 70, pre-post correlation = 0.4) is 160 per group, spread across 8 clusters each. This sample size provides adequate power (≥90%) to detect minimum differences of interest on secondary workplace and overall activity outcomes, with assumed SDs and pre-post correlations (r) of 70–75 mins (r = 0.4) for sitting, standing and prolonged sitting; 20–35 minutes (r = 0.7) for stepping and MVPA; and, 60 minutes (r = 0.7) for light activity.

For the other secondary outcomes, minimum detectable differences (MDD) with 80-90% power were: 1.7-1.8 kg weight, 1.3-1.5 kg lean body mass and fat mass, 1.5-1.7 cm waist circumference, 5–6 mmHg systolic blood pressure, 4 mmHg diastolic blood pressure, 11–13 pmol insulin, 0.28-0.33 mmol/L glucose, 0.24-0.25 /0.19-0.22 / 0.10-0.11 mmol/L total / LDL- / HDL- cholesterol, 0.12-0.14 mmol/L log triglycerides, 0.3-0.4 units work performance, 0.3-0.4 units eyestrain, 0.6-0.7 units fatigue. Based on these MDDs, power would be adequate to detect effect sizes of the magnitude observed in the pilot study [20] for glucose (0.3 mmol/L), insulin (15.7 pmol) log triglycerides (0.19 mmol/L) and diastolic blood pressure (4.0 mmHg) only.

### Statistical analyses

In accordance with the study aims, statistical analyses will be conducted to determine whether the intervention group differs from the control group in changes over time in primary and secondary outcomes. Statistical significance will be set at the conventional 5% level (two-tailed). Consistent with the cluster-randomised design, and in order to examine both workplace-level and individual-level variation, Linear Mixed Models will be used (SAS version 9.3 or STATA version 12). These models will use random intercepts for workplace and individuals (to account for clustering within workplaces and repeated measures) and will adjust for baseline values and potential confounders. Appropriate distributions (e.g., normal, gamma, binomial) and links (e.g., identity, log) will be used depending on the distribution of the data. *Potential confounders* will initially be identified *a priori* based on the findings of our preceding epidemiological studies and the relevant research literature, and will be controlled via statistical adjustment as relevant to each specific outcome should they display association with the outcome (significant at p < 0.2). Analyses will follow intention-to-treat principles. Assumptions will be tested regarding missing data, randomisation, and contamination (an important consideration given that the study sites are all within the one organisation). Contamination will be assessed by tracking individuals within study sites (i.e. transfers to a study site of the same allocation, no allocation or opposite allocation) and by assessing, at the end of the program, self-reported use of specific intervention strategies to reduce prolonged sitting.

#### *Moderation and mediation*

Moderator analysis will examine whether intervention effects differ across individual (e.g. age, gender) and workplace characteristics (e.g. office layout). Moderation will be tested using multilevel models (as above) with interaction terms to test moderation. Theoretically-driven constructs and mechanisms (described above) will be examined as possible mediators of the intervention effects, using established methods appropriate to a cluster design as some proposed mediators will vary at the individual-level (e.g. preferences) whilst others will only vary at the organisational level (e.g. manager’s attitudes and knowledge) [73].

#### *Incremental cost-effectiveness analyses*

Detailed pathway analysis will be used to specify all activities undertaken as part of the intervention in order to measure costs of associated resource use (e.g. provision and installation of workstations, information sessions, telephone check-ups, weekly emails, adverse events). Unit costs will be drawn from best available sources for the 2012 reference year. In addition to incremental costs of the intervention (measured against the comparator), incremental cost offsets attributable to disease prevention in the long-term will be reported. The cost data will be combined with the behavioural and biomarker outcomes to produce a range of incremental cost effectiveness ratios (ICERs), across both primary and secondary outcomes, including cost per unit reduction in sedentary time and cost per quality-adjusted life year (QALY) gained (based on AQoL-8D [72]). The modelled evaluation will use a Markov approach to estimate the health and cost impacts of changes in sedentary status over the lifetime of participants. Standard discounting will be applied to both costs and outcomes. Simulation-modelling using the @RISK software package will be employed to calculate 95% uncertainty intervals (median, 2.5 and 97.5 percentiles) around the epidemiological probabilities and cost estimates.

## Discussion

Substantial epidemiological evidence has provided the rationale for identifying prolonged workplace sitting time as an emerging public health concern. The critical next step in informing public health policy and practice is the conduct of randomised controlled workplace intervention trials. Such trials are essential for determining the feasibility, effectiveness and sustainability of reducing workplace sitting time, as well as the impact on biomarkers of chronic disease risk. The *Stand Up Victoria* trial is unique, in that it takes a whole-of-organisation approach to reducing prolonged workplace sitting, evaluates the impact of the intervention on a broad range of health (including biomarkers of cardiovascular health) and work-related outcomes, and incorporates objective measures of workplace sitting time and physical activity – as distinct from relying on self-report measures. It will also measure the cost effectiveness of the intervention – a critical influence in deciding future uptake within workplaces. Similarly, identification of the moderators of the intervention effect on workplace sitting will lead to improved understanding of which workers may be most suited to this type of intervention. These analyses may help inform targeted delivery of the intervention to specific sub-groups of workers and the appropriate adaptation of the intervention for other sub-groups for which it was less successful. These methodological strengths are important for advancing the science of settings-based approaches to sedentary behaviour change, as well as building the evidence base for the translation of this work into population-health and workplace-health practice.

## Competing interests

Ergotron Pty Ltd (http://www.ergotron.com) has previously provided workstations for formative research related to the topic. Dunstan presented at the 'JustStand Wellness Summit', a conference organised by Ergotron, in 2012 and Healy presented at the same summit in 2013. Ergotron covered travel and accommodation expenses for both Dunstan and Healy. No further honoraria or imbursements were received.

## Authors’ contributions

DD, GH, EE, NO, AL, MM conceived the study and subsequently obtained funding from the National Health and Medical Research Council and the Victorian Health Promotion Foundation. DD, GW, EE, NO, AL, MM, MN, EW, BJ, SL and GH participated in the design and coordination of the methodology and measurement tools and helped to draft the manuscript. EW provided expert input on statistical analyses. MM wrote the section related to the economic evaluation. All authors read and approved the final manuscript.

## Pre-publication history

The pre-publication history for this paper can be accessed here:

http://www.biomedcentral.com/1471-2458/13/1057/prepub

## Supplementary Material

Additional file 1: Figure S1 Information booklet. Click here for file

Additional file 2: Figure S2 Management email template example. Click here for file

Additional file 3: Figure S3 Workstation tip sheet. Click here for file

Additional file 4: Figure S4 Example of baseline feedback letter. Click here for file

Additional file 5: Figure S5 Workstation tracker. Click here for file

Additional file 6: Figure S6 Questionnaire items regarding adequacy of work space and working with colleagues. Click here for file

Additional file 7: Table S1 Psychometric properties of the mediator scales used in the Stand Up Victoria study. Click here for file

Additional file 8: Figure S7 Mediator/Moderator questions. Click here for file

Additional file 9: Figure S8 Adverse Events. Click here for file
