# Supplementary material for: Reducing office workers’ sitting time: rationale and study design for the Stand Up Victoria cluster randomized trial
Source: BMC Public Health. 2013 Nov 9;13:1057. doi: 10.1186/1471-2458-13-1057 (PMC3828481; doi:10.1186/1471-2458-13-1057)
Supplement: Additional file 1: Figure S1 — Information booklet. [file 1471-2458-13-1057-S1.pdf]

STAND UP • SIT LESS • MOVE MORE

Workplace Sitting and Health

The health benefits of regular exercise have been well known for several decades. However, new research has shown that less sitting is related to a lower risk of developing type 2 diabetes, lower risk of premature death from heart disease and a decreased risk of developing some cancers. Sitting less is also known to positively influence risk factors which contribute to diabetes and heart disease such as body size and blood sugar levels. Changes in the types of jobs we do, the way we travel and how we spend our leisure time mean that we are sitting throughout the day more now than ever before. Importantly, the negative impacts on health of too much sitting have been found **even in people who regularly exercise.**

These scientific findings do not mean that everybody who sits for long periods at work will develop these diseases. We are all different, and our health is determined by our family history, our lifestyle, and by a wide range of other influences. Nevertheless, reducing sitting time at work will have a range of benefits for your health and well-being.

"Stand Up Victoria" – A Workplace Study

Australian office workers sit for about 75% of their work hours, or about 6 hours of an 8-hour shift. You have been fitted with a small activity monitor, which records how often and how long *you* spend standing up, sitting down, and moving about. You will get feedback on your activity patterns during your individual consultation session that will occur in the next two weeks.

The "Stand Up Victoria" Study is a program that supports you to **stand up, sit less and move more** at work. We will provide this support by installing a new sit-stand-workstation for you to use and by talking to you about practical ways to help you stand up, sit less and move more at work. By doing so, you will increase the time that you are more active (both standing and moving about), which may help to make you:

- feel better
- have more energy
- be able to concentrate for longer periods of time and
- be more productive

Stand Up, Sit Less, Move More – Setting Your Goals

Many people find that setting themselves specific goals helps to change habits. In doing so, it is important to start with a less ambitious and more achievable goal- then you can gradually build this up over time. Over the next month our consultants will help you to figure out practical strategies that work for you. Below we have outlined the three key recommendations that we will support you to reach in the "Stand Up Victoria" program.

1) Stand up more often

The first recommendation is to change your posture regularly and *break up long periods of sitting.* This can be as simple as quickly standing up at your desk, having a little stretch, and sitting back down again. We recommend that you try to *stand up at least once every 30 minutes.* You may find it helpful to use some prompts to regularly remind you to stand up (e.g. stand up every time the phone rings or someone walks into your office). We have attached a list of strategies you could use at the end of this information booklet and will help you identify some that work for you during the consultation session with our health coach.

2) Sit Less

The second recommendation is about *reducing your overall sitting time.* This is where you can make use of your new workstation. It allows you to do a lot of your work tasks in a standing position rather than sitting at your desk. You could also stand during meetings, presentations, or workshops. As a general guideline, we recommend sitting and standing at your workstation in equal proportions throughout the day. But this will vary for each person, with some doing more and some doing less. For most people, it will take some time to build up to standing more. The most important thing is to listen to your body and to alter you posture regularly. Try to stand up every 30 min, even if it is just standing up and sitting directly back

down AND, when you can, try standing at your workstation for as long as you are comfortable, but probably not for more than half an hour, at least to start.

At first, you may find it challenging to change the way you're used to working, but we will assist you to develop new habits that suit you and your work patterns. By replacing your sitting with standing up, you are making your muscles work harder, which can lead to better heart health.

3) Move More

The third recommendation is about *moving more* at work. This strategy will aim to not only get you to stand up but to *move around your work space more*. There are lots of strategies that we will show you to help you move more. By moving more you will increase your daily energy expenditure, which can help improve your health.

Next Steps

We thank you for taking part in the "Stand Up Victoria" Study. We hope you enjoy your new workstation and being more active at your workplace. One of our consultants will be installing your new sit-stand workstation and arranging your individual consultation session in the next week (or may even have already done so). On the next page you can see an overview of the different times that we will be in contact with you.

In the mean time if you have any questions please don't hesitate to contact us.

Project Coordinator:

Glen Wiesner

Email: Glen.Wiesner@bakeridi.edu.au

Stand Up Victoria - Study Timeline

Time point	Activity
Assessment 1 (Baseline)	Blood & Body Measures
	Questionnaires
	Wearing Monitor & filling in a diary for 1 week
Week 1	Group Information session & Email summary
	Workstation Installation and check-in by Glen
	Individual Consultation & Email summary
Week 2	Telephone check-in 1
Week 4	Telephone check-in 2
Week 8	Telephone check-in 3
Week 12	Telephone check-in 4
Assessment 2 (3 months)	Blood & Body Measures
	Questionnaires
	Wearing Monitor & filling in a diary for 1 week
Assessment 3 (12 months)	Blood & Body Measures
	Questionnaires
	Wearing Monitor & filling in a diary for 1 week
12 months	Feedback

Strategies to STAND UP more often

- ☐ Set timer (e.g. online: www.online-stopwatch.com or through MS Outlook)
- ☐ When your phone rings/ every time you call someone (put phone upon a shelf or wear a headset)
- ☐ After completing a task
- ☐ When you get tired
- ☐ When you feel discomfort in your back/neck/ shoulders
- ☐ When someone enters your office/ workspace
- ☐ During meetings/ presentations
- ☐ When you see a colleague stand up

Add your own or organisation specific strategies on the lines below:

- ☐ ____
- ☐ ____
- ☐ ____
- ☐ ____

Strategies to Move More

- ☐ iMails (walk over and talk) instead of eMails to colleagues
- ☐ Remove bins/printers from your office and use a central one
- ☐ Dispose of waste/ collect printing more frequently
- ☐ Drink more water so you have to go to the water cooler (and bathroom!) more often
- ☐ Use a bathroom that is further away
- ☐ Step outside for fresh air
- ☐ Use the stairs instead of the lift
- ☐ Use an active way of commuting to work (walk or ride your bike; stand up in train; stand up to wait for your train/bus; ...)
- ☐ Park your car further away from your workplace and have a short walk or park in short term parking
- ☐ Have lunch away from your desk

Add your own or organisation specific strategies on the lines below:

- ☐ ____
- ☐ ____
- ☐ ____
