# Supplementary material for: Reducing office workers’ sitting time: rationale and study design for the Stand Up Victoria cluster randomized trial
Source: BMC Public Health. 2013 Nov 9;13:1057. doi: 10.1186/1471-2458-13-1057 (PMC3828481; doi:10.1186/1471-2458-13-1057)

STAND UP – SIT LESS – MOVE MORE

Dear Team,

This is **Week 2** of the Stand Up VIC study. I hope you are finding your new workstation helpful to stand more and are enjoying a healthier way to work!

[OPTIONAL: e.g.: During my walk through the office this morning I have noticed that many of you have been standing. Great effort team!]

Here is your STAND UP – SIT LESS – MOVE MORE TIP OF THE WEEK:

Drink more water! It has the dual benefit of keeping you hydrated and encourages you to move to and from the tea room and bathroom.

Keep standing,

[Management Signature]

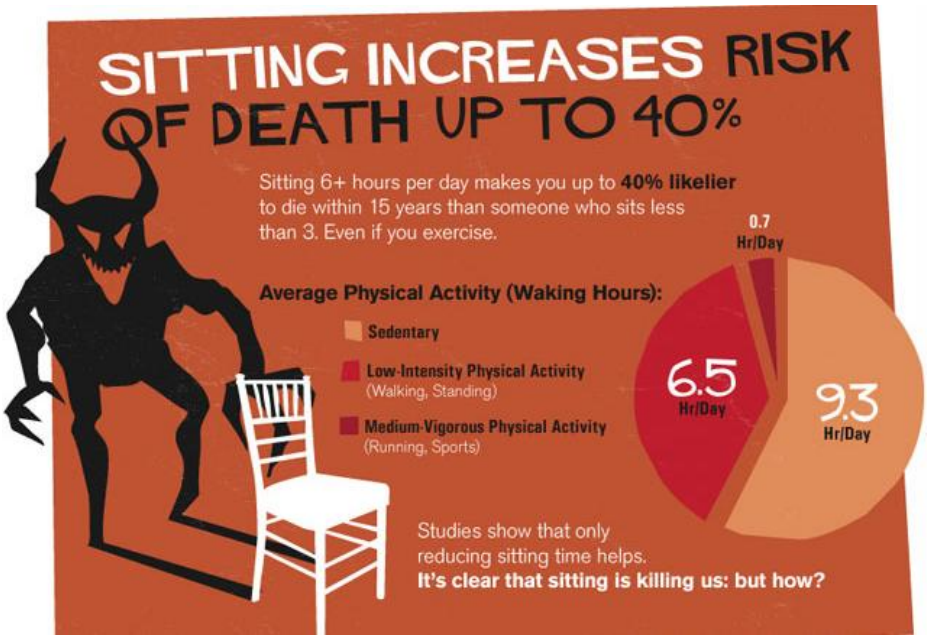

Supplement: Additional file 2: Figure S2 — Management email template example. [file 1471-2458-13-1057-S2.pdf]
