# Supplementary material for: Reducing office workers’ sitting time: rationale and study design for the Stand Up Victoria cluster randomized trial
Source: BMC Public Health. 2013 Nov 9;13:1057. doi: 10.1186/1471-2458-13-1057 (PMC3828481; doi:10.1186/1471-2458-13-1057)
Supplement: Additional file 3: Figure S3 — Workstation tip sheet. [file 1471-2458-13-1057-S3.pdf]

STAND UP • SIT LESS • MOVE MORE

Top Tips for Using your Workstation

1. Position the screen height to be within 15-50 degrees below your horizontal line of vision. It is preferable to use reading glasses or progressive lenses that enable you to view the screen through the mid range of the lens. If using bifocals, you may need to lower the monitor further in order to attain neutral head and neck position.
2. The top of your keyboard should be approximately level with the height of your elbow.
3. Avoid elevating the back aspect of the keyboard as this will result in upward bending of the wrists. You may wish to use a soft gel wrist rest to provide wrist support in the case that there is adequate space.
4. Arrange the materials and equipment used at your workstation, in a way that enables you to stand periodically during computer use.
5. Prolonged periods of standing and sitting can result in muscle fatigue and accordingly you are encouraged to change between seated and standing postures by adjusting the overall height of the workstation. The height difference between the screen and the keyboard is likely to remain constant. Remember that when seated in the chair and when standing, the height of your elbow should approximate the height of the keyboard. The seat height may require adjustment to achieve this.
6. You are encouraged to change between seated and standing postures at the computer workstation, on a half-hourly basis to start, building up to longer standing periods over time. In the case that you experience discomfort or pain you should change your posture.
7. If you cannot stand for 30 minutes at a time, try to stand in shorter bouts (e.g. 10-minute bouts throughout the day).
8. You are changing a habit which you have probably had for many years (i.e. sitting down for most of your time at work) – make small changes, and always listen to your body and change posture or sit down if you need to.
9. Ladies may like to keep a pair of comfortable, flat-soled shoes near their desk to wear when standing for prolonged periods.
10. When standing, consider resting one foot forward and slightly elevated (e.g. on a phonebook) to improve comfort. Alternate the leading foot as required.

The images below depict optimal computer workstation adjustment between sitting and standing:

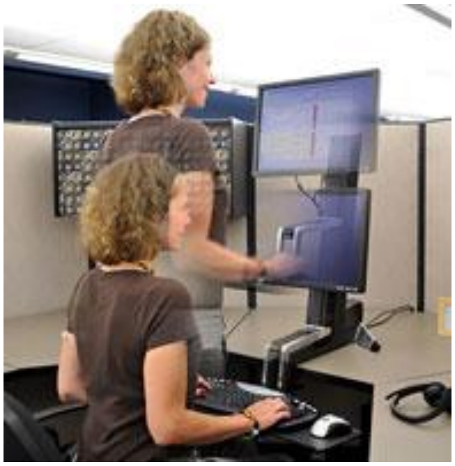

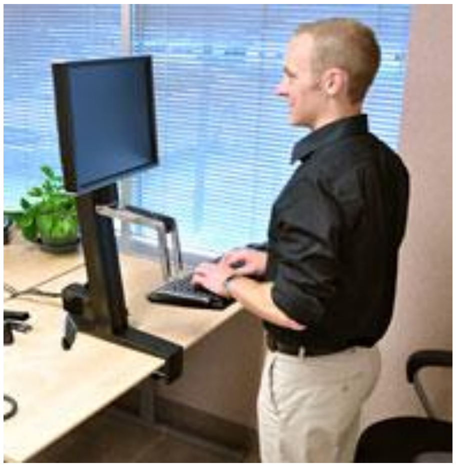

Source: http://www.ergotron.com/Products/tabid/65/PRDID/379/language/en-AU/Default.aspx

Stand UP VIC

STAND UP • SIT LESS • MOVE MORE

Workstation Information Sheet

To determine the appropriate values below for an optimal workstation adjustment, simply enter your height on: http://www.ergotron.com/tabid/305/language/en-AU/Default.aspx

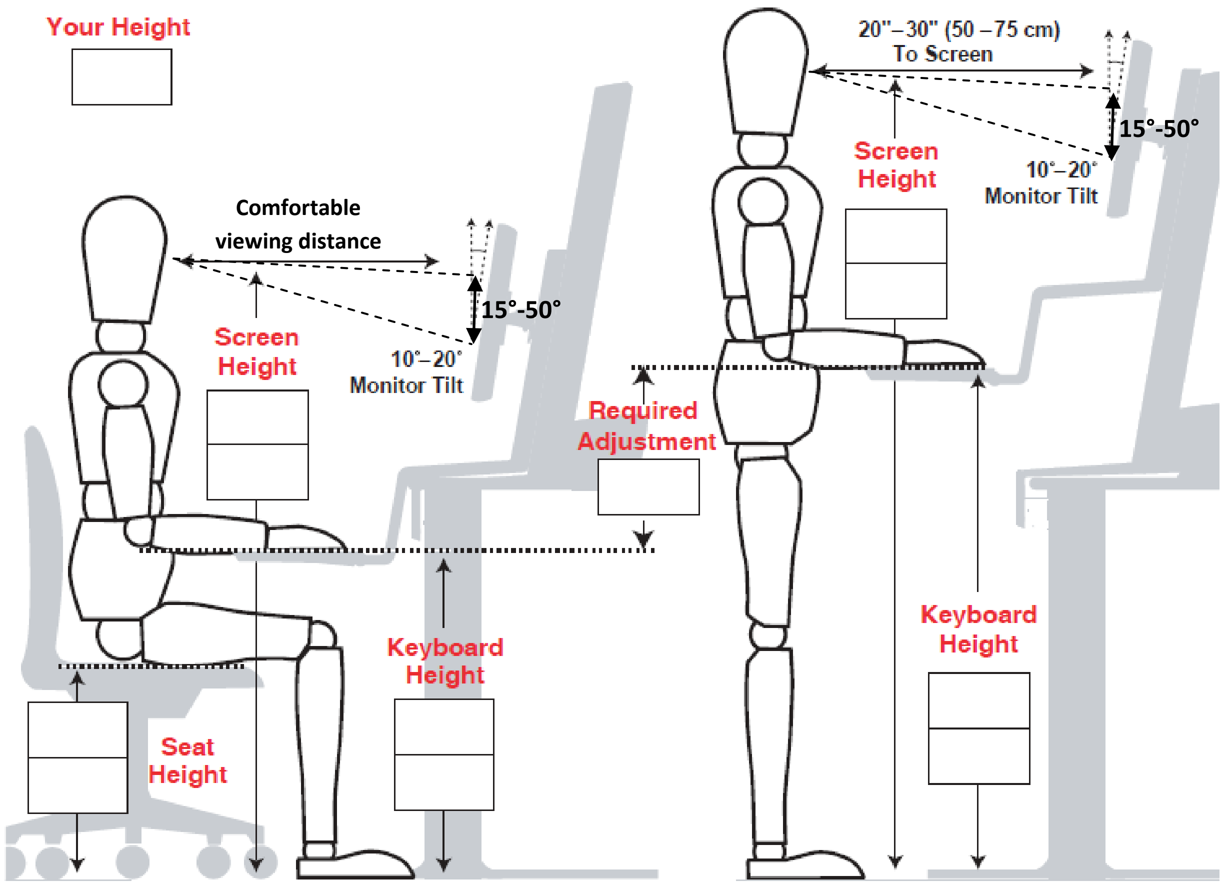

Source: http://www.ergotron.com/tabid/305/language/en-AU/Default.aspx

Note: The values represent average dimensions for people of your stature and do not account for variations due to gender, age or body type. Refer to the values as a starting point, rather than the final mounting height of your computer equipment. Values are derived without clothing allowances. Always add shoe height to figure proper measurement. Additional factors may apply. Consult with an ergonomist for more detailed information. Values are based on the 1988 Anthropocentric Survey of the U.S. Army Personnel database.

For more product information, please refer to the manufacturer website at: http://www.ergotron.com
