# Supplementary material for: Reducing office workers’ sitting time: rationale and study design for the Stand Up Victoria cluster randomized trial
Source: BMC Public Health. 2013 Nov 9;13:1057. doi: 10.1186/1471-2458-13-1057 (PMC3828481; doi:10.1186/1471-2458-13-1057)
Supplement: Additional file 4: Figure S4 — Example of baseline feedback letter. [file 1471-2458-13-1057-S4.pdf]

STAND UP • SIT LESS • MOVE MORE

Personal Feedback Report – Assessment 1

Dear John (ID: XXX),

Thank you for completing Assessment 1 of the Stand Up Victoria study.

Please find enclosed your personal feedback report, which summarises information about your sitting, standing, and moving time at and outside of your primary workplace.

Thanks again for your participation in the study.

If you have any questions or concerns, please do not hesitate to contact us.

Kind regards,

Posture and Movement

Below is a summary of the information that we obtained from the activity monitors that you wore for the seven day assessment. For each of the figures, red is time sitting, yellow is time standing, and green is time moving.

At the workplace

The following figures summarize information about your posture and movement while you were wearing the thigh monitor at your workplace. The pie chart shows the proportion (in percent) of time at your primary workplace spent sitting (in bouts of 30 mins or longer at a time and in bouts of less than 30 mins at a time), standing, and stepping. These figures do not include times you removed your monitors or times you spent working at locations other than your primary workplace.

Average proportion of time at the workplace spent sitting (bouts ≥30min & <30mins), standing and moving

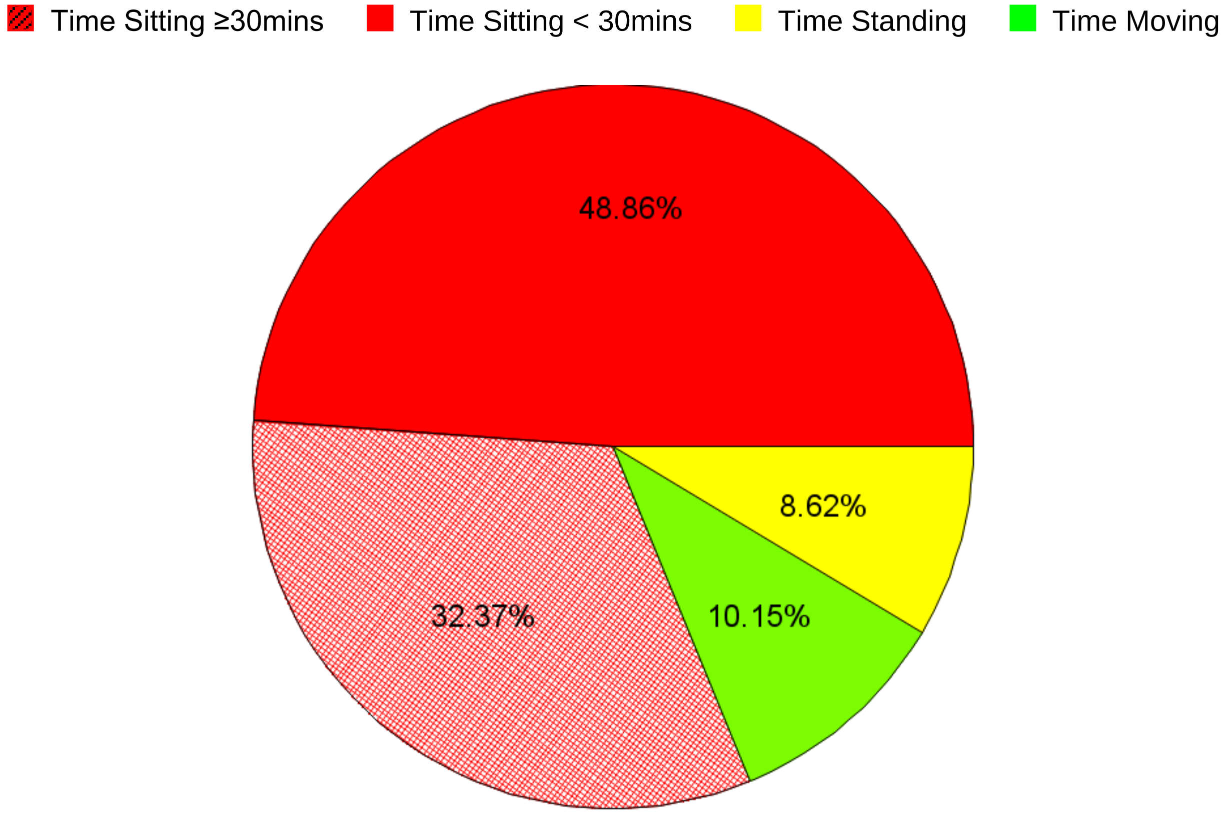

**Assessment 1:**	**24/08/2013 - 30/08/2013**
**Average work time:**	**7.8 hours per day**
**Average number of sitting bouts ≥30mins:**	**3 per day**

During all your waking hours

The following figures summarize information about your posture and movement while you were awake.

1) The pie chart shows the percentage of your waking hours that you spent sitting (in bouts of 30 mins or longer at a time and in bouts of less than 30 mins at a time), standing, and moving. These figures do not include the times you were asleep or any times you may have removed your monitor.

Average proportion of waking time between 6am & 10pm spent sitting (bouts ≥30min & <30mins), standing and moving

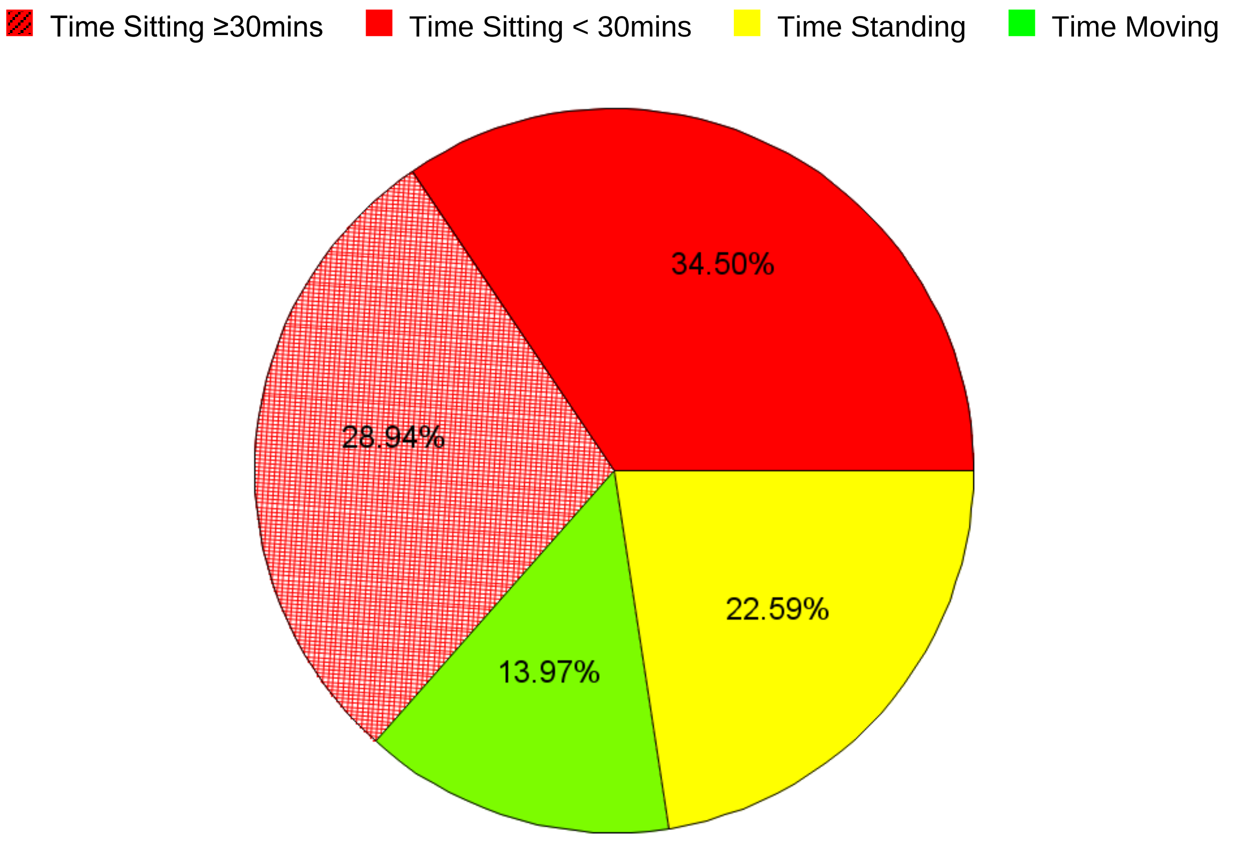

Assessment 1: 24/08/2013 - 30/08/2013

Average number of sitting bouts ≥30mins: 6 per day

2) The following figure, a *heat-map*, is useful for understanding your activity pattern, because it shows when you performed each activity, and any times that you might be performing the same activity for an extended period without interruption. The activity that your monitor recorded each day is shown in each column – from the beginning of the day (bottom) to the end of the day (top). Each coloured block or line represents the activity the monitor recorded at that particular time, on that particular day (sitting-red, standing-yellow or moving-green). Any times you were asleep or not wearing the monitor are not coloured.

Assessment 1: Daily patterns of time spent sitting, standing and moving

Time Sitting   Time Standing   Time Moving

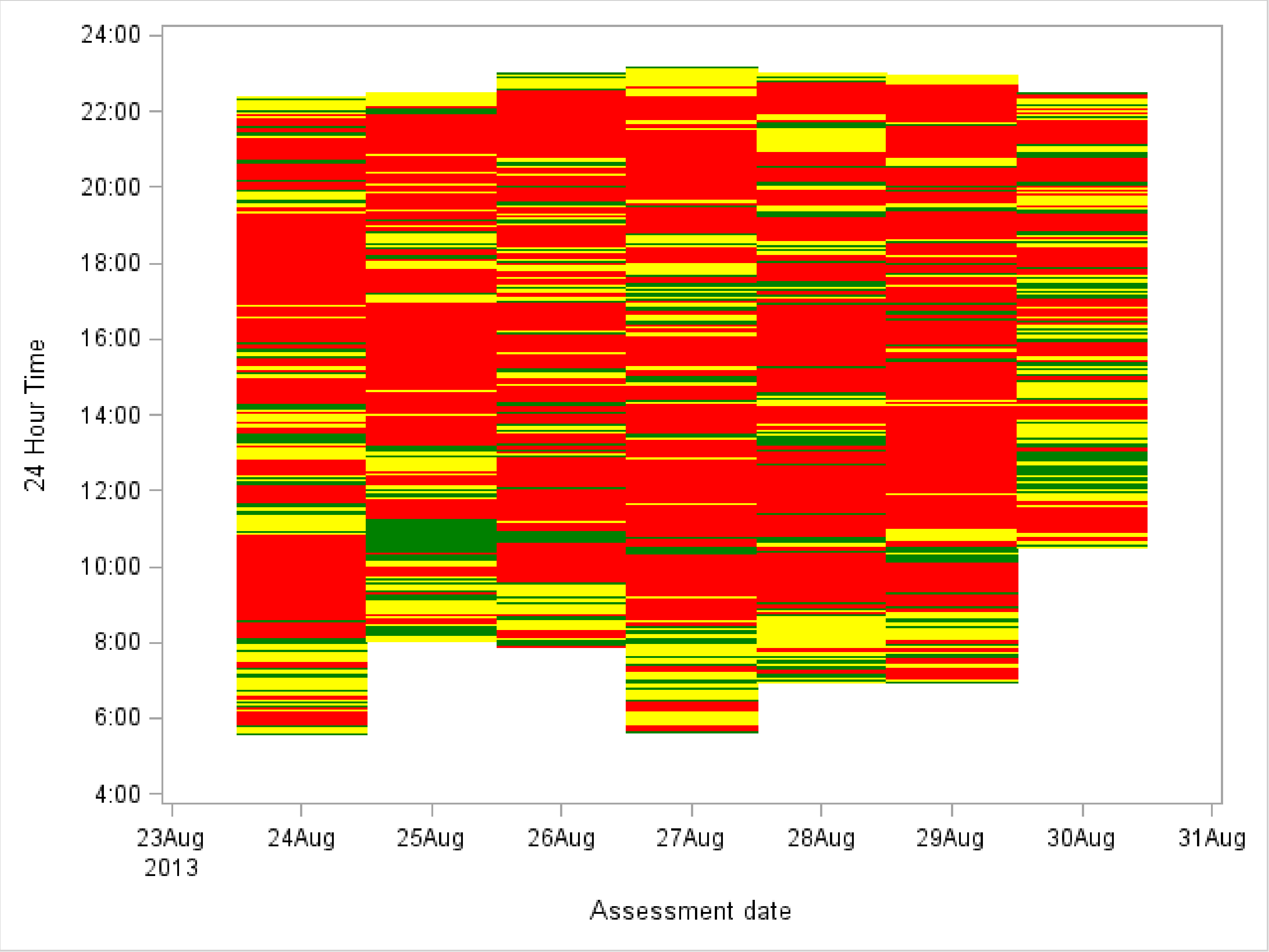

You were at your workplace:

26/08/2013 9:35 -17:05   27/08/2013 9:00 -16:15   28/08/2013 9:00 -17:15   29/08/2013 9:00 -17:25
