# Supplementary figures and images for: Reducing office workers’ sitting time: rationale and study design for the Stand Up Victoria cluster randomized trial
Source: BMC Public Health. 2013 Nov 9;13:1057. doi: 10.1186/1471-2458-13-1057 (PMC3828481; doi:10.1186/1471-2458-13-1057)

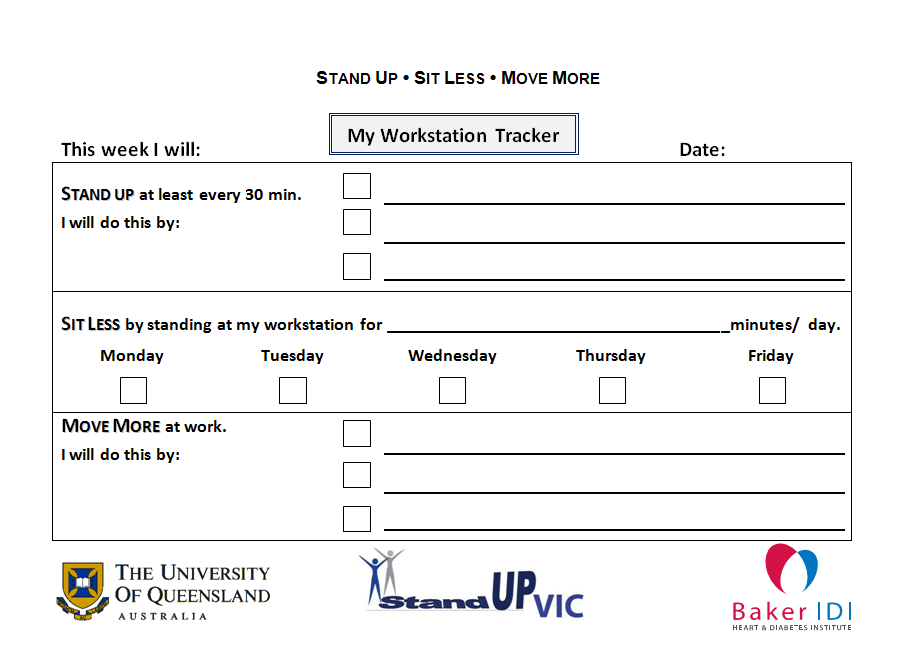

Supplement: Additional file 5: Figure S5 — Workstation tracker. [file 1471-2458-13-1057-S5.png]
