# Supplementary material for: Reducing office workers’ sitting time: rationale and study design for the Stand Up Victoria cluster randomized trial
Source: BMC Public Health. 2013 Nov 9;13:1057. doi: 10.1186/1471-2458-13-1057 (PMC3828481; doi:10.1186/1471-2458-13-1057)
Supplement: Additional file 6: Figure S6 — Questionnaire items regarding adequacy of work space and working with colleagues. [file 1471-2458-13-1057-S6.pdf]

Office environment: interactions at work and adequacy of space

1. **How often did you interact with others at work in the ways described below? Please estimate the frequency and average duration of:**

Formal meetings: pre-scheduled meetings with colleagues and external parties, including telephone and video conferences.

Informal discussions: ad hoc meeting or conversation (not necessarily related to work) taking place away from your desk (e.g., in work space, lounge, printer/copier area, corridor, kitchen, tea room).

Collaborative working: working together with colleagues over something while sharing the same space.

Please do NOT include having lunch with others in the above categories.

	Times in last 7 days	Average duration (min)
Formal meetings		
Informal discussions		
Collaborative working		

2. **Below are questions about spaces for formal meetings, informal discussion and collaborative working.**

For each of the questions below, please choose the response that best applies to your workplace. When considering informal discussion spaces, please think about spaces away from your desk. Please note that "functionally adequate" refers to quality aspects (e.g., equipment, furnishing, lighting, comfort, etc).

	Strongly disagree	Disagree	Neither disagree nor agree	Agree	Strongly agree	NA
There is a sufficient amount of space for formal meetings in my workplace.	☐	☐	☐	☐	☐	☐
The space for formal meetings is functionally adequate. (Select NA if no such space exists.)	☐	☐	☐	☐	☐	☐
There is a sufficient amount of space for informal discussion in my workplace.	☐	☐	☐	☐	☐	☐
The space for informal discussion is functionally adequate. (Select NA if no such space exists.)	☐	☐	☐	☐	☐	☐
There is a sufficient amount of space for collaborative working in my workplace.	☐	☐	☐	☐	☐	☐
The space for collaborative working is functionally adequate. (Select NA if no such space exists.)	☐	☐	☐	☐	☐	☐

The above materials have been developed for use within the Stand Up Victoria study. Any future use of these materials must be referenced to this article.
