# Supplementary material for: Reducing office workers’ sitting time: rationale and study design for the Stand Up Victoria cluster randomized trial
Source: BMC Public Health. 2013 Nov 9;13:1057. doi: 10.1186/1471-2458-13-1057 (PMC3828481; doi:10.1186/1471-2458-13-1057)
Supplement: Additional file 7: Table S1 — Psychometric properties of the mediator scales used in the Stand Up Victoria study. [file 1471-2458-13-1057-S7.docx]

**Supplementary Table 1:** *Psychometric properties of the mediator scales used in the Stand Up Victoria study - data from previous pilot of workplace sitting intervention [20].*

| **Construct and scale items** | **Internal Consistency (Cronbach’s Alpha)** | **Test-retest reliability (Spearman’s rho)** | **Proportion of sample with missing items** |
| --- | --- | --- | --- |
| **Preference for sitting and standing at work** | (n=40; both groups) | (n=19; control group only) | (n=40; both groups) |
| 1. If you were given a choice, how much time would you like to spend sitting at work? | Not applicable | 0.67 | 0% |
| 1. If you were given a choice, how much time would you like to spend standing up at work? |  | 0.78 |  |
| **Knowledge** |  |  |  |
| 1. Sitting for most of the time at work does not impact on my health *(NB: reverse scored)* | 0.48 | 0.76 | 0% |
| 1. Sitting for most of the time at work is bad for my health |  |  |  |
| 1. Any health impact of sitting for most of the time at work can be off-set by exercising at other times of the day *(NB: reverse scored)* |  |  |  |
| 1. It is beneficial for my health to stand up at least once every 30 minutes while I am at work |  |  |  |
| 1. It is beneficial for my health if I am as active as possible throughout my working day (e.g. by using the stairs instead of the lift) |  |  |  |
| **Barrier self-efficacy** |  |  |  |
| 1. Stood up during meetings at work, even though no one else was | 0.90 | 0.66 | 0% |
| 1. Stood up during meetings at work, even when supervisors were sitting down |  |  |  |
| 1. Stood up at your desk at work, even though your colleagues were not |  |  |  |
| 1. Stood up at your desk at work, even when you felt tired |  |  |  |
| 1. Stood up at your desk at work, even if your footwear was uncomfortable |  |  |  |
| 1. Stood up at your desk at work, even though you were really busy at work |  |  |  |
| 1. Stood up at your desk at work , even when your tasks required looking at multiple papers |  |  |  |
| 1. Stood up at your desk at work , even when your tasks required talking on the phone |  |  |  |
| 1. Walk to talk to a colleague at work instead of emailing them, even though others didn’t |  |  |  |
| **Perceived behavioural control** |  |  |  |
| 1. It is my choice whether I stand up or sit at my desk while at work | 0.81 | 0.55 | 0% |
| 1. It is my choice whether I stand up or sit during a meeting with colleagues at work |  |  |  |
| 1. It is my choice whether I stand up or sit during a meeting with my supervisor/s at work |  |  |  |
| 1. It is my choice whether I walk over to talk to a colleague (*iMail)* or send them an eMail |  |  |  |
| 1. It is my choice whether I walk over to talk to a supervisor (*iMail)* or send them an eMail |  |  |  |
| **Perceived organisational social norms** |  |  |  |
| 1. My workplace is committed to supporting staff health and well-being | 0.90 | 0.60 | 0% |
| 1. My workplace is committed to supporting staff choices to stand or move more at work |  |  |  |
| 1. My colleagues would not mind if I chose to stand up while working at my desk |  |  |  |
| 1. My supervisor/s would not mind if I chose to stand up while working at my desk |  |  |  |
| 1. My colleagues would not mind if I chose to stand during a work meeting |  |  |  |
| 1. My supervisor/s would not mind if I chose to stand during a work meeting |  |  |  |
| 1. My colleagues would not mind if I chose to walk over and talk to them (*iMail*) rather than sending them an eMail |  |  |  |
| 1. My supervisor/s would not mind if I chose to walk over and talk to them (*iMail*) rather than sending them an eMail |  |  |  |
| **Self-regulation strategies** |  |  |  |
| 1. Thought about how much I sit at work | 0.84 | 0.57 | 0% |
| 1. Recorded my sitting or standing at work in a written record |  |  |  |
| 1. Paid attention to specific things to help me stand at work (e.g., I have more energy in the morning so I stand during this time) |  |  |  |
| 1. Set short-term goals (daily or weekly) related to how often I stand up at work |  |  |  |
| 1. Broken down larger goals into smaller, more manageable goals (e.g. accumulate 40 minutes of standing in 4 x10minute bouts) |  |  |  |
| 1. Thought about my standing goals |  |  |  |
| 1. Reminded myself of the health benefits of standing at work (e.g., reduced risk of Type 2 diabetes, premature death) |  |  |  |
| 1. Scheduled specific times to stand up at work |  |  |  |
| 1. Paid attention to barriers which got in the way of my standing at work |  |  |  |
| 1. Planned ways to overcome barriers to my standing at work |  |  |  |
| **Intervention-specific strategies** |  |  |  |
| 1. Moved my workstation from sitting to standing or vice versa | 0.91 | 0.66 | 0% |
| 1. Used my workstation tracker to record the days that I reach my standing goal at work |  |  |  |
| 1. Used my workstation tracker to record my strategies for standing up and moving more at work |  |  |  |
| 1. Used an on-line timer to time my periods of sitting or standing |  |  |  |
| 1. Stood up when someone approached me at my workstation |  |  |  |
| 1. Stood up when my phone rings |  |  |  |
| 1. Walked to talk to a colleague (*iMail)* rather than sending them an eMail |  |  |  |
| 1. Walked to the printer that is further away from my workstation |  |  |  |
| 1. Walked to the bathroom that is further away from my workstation |  |  |  |
