# Supplementary material for: Reducing office workers’ sitting time: rationale and study design for the Stand Up Victoria cluster randomized trial
Source: BMC Public Health. 2013 Nov 9;13:1057. doi: 10.1186/1471-2458-13-1057 (PMC3828481; doi:10.1186/1471-2458-13-1057)
Supplement: Additional file 8: Figure S7 — Mediator/Moderator questions. [file 1471-2458-13-1057-S8.pdf]

***For the following questions, we would like you to think how you felt in the past month. Please answer the following questions by ticking the appropriate box.***

**1. Tick the box that best applies to you:**

| | | None of the time | 10-30% of the time | 30-50% of the time | 50-80% of the time | 80-100% of the time |
|---|---|---|---|---|---|---|
| a. | if you were given a choice, how much time would you like to spend sitting at work? | ☐ | ☐ | ☐ | ☐ | ☐ |
| b. | if you were given a choice, how much time would you like to spend standing up at work? | ☐ | ☐ | ☐ | ☐ | ☐ |

**2. Please indicate how much you agree or disagree with the following statements:**

| | | Strongly Disagree | Disagree | Not Sure | Agree | Strongly Agree |
|---|---|---|---|---|---|---|
| a. | sitting for most of the time at work does not impact on my health | ☐ | ☐ | ☐ | ☐ | ☐ |
| b. | sitting for most of the time at work is bad for my health | ☐ | ☐ | ☐ | ☐ | ☐ |
| c. | any health impact of sitting for most of the time at work can be off-set by exercising at other times of the day | ☐ | ☐ | ☐ | ☐ | ☐ |
| d. | it is beneficial for my health to stand up at least once every 30 minutes while I am at work | ☐ | ☐ | ☐ | ☐ | ☐ |
| e. | it is beneficial for my health if I am as active as possible throughout my working day (e.g. by using the stairs instead of the lift) | ☐ | ☐ | ☐ | ☐ | ☐ |

**3. Please indicate how much you agree or disagree with the following statements:**

| | Strongly Disagree | Disagree | Not Sure | Agree | Strongly Agree |
|---|---|---|---|---|---|
| a. it is my choice whether I stand up or sit at my desk while at work | ☐ | ☐ | ☐ | ☐ | ☐ |
| b. it is my choice whether I stand up or sit during a meeting with colleagues at work | ☐ | ☐ | ☐ | ☐ | ☐ |
| c. it is my choice whether I stand up or sit during a meeting with my supervisor/s at work | ☐ | ☐ | ☐ | ☐ | ☐ |
| d. it is my choice whether I walk over to talk to a colleague (*iMail)* or send them an eMail | ☐ | ☐ | ☐ | ☐ | ☐ |
| e. it is my choice whether I walk over to talk to a supervisor (*iMail)* or send them an eMail | ☐ | ☐ | ☐ | ☐ | ☐ |

**4. Please indicate how much you agree or disagree with the following statements:**

| | Strongly Disagree | Disagree | Not Sure | Agree | Strongly Agree |
|---|---|---|---|---|---|
| a. my workplace is committed to supporting staff health and well-being | ☐ | ☐ | ☐ | ☐ | ☐ |
| b. my workplace is committed to supporting staff choices to stand or move more at work | ☐ | ☐ | ☐ | ☐ | ☐ |
| c. my colleagues would not mind if I chose to stand up while working at my desk | ☐ | ☐ | ☐ | ☐ | ☐ |
| d. my supervisor/s would not mind if I chose to stand up while working at my desk | ☐ | ☐ | ☐ | ☐ | ☐ |
| e. my colleagues would not mind if I chose to stand during a work meeting | ☐ | ☐ | ☐ | ☐ | ☐ |

| | | | | | | |
|---|---|---|---|---|---|---|
| f. | my supervisor/s would not mind if I chose to stand during a work meeting | ☐ | ☐ | ☐ | ☐ | ☐ |
| g. | my colleagues would not mind if I chose to walk over and talk to them (*iMail*) rather than sending them an eMail | ☐ | ☐ | ☐ | ☐ | ☐ |
| h. | my supervisor/s would not mind if I chose to walk over and talk to them (*iMail*) rather than sending them an eMail | ☐ | ☐ | ☐ | ☐ | ☐ |

**5. The next questions are about how often you used various strategies to help you stand up at work. In the *past month* I have:**

| | | Never | Rarely | Some-times | Often | Very Often |
|---|---|---|---|---|---|---|
| a. | thought about how much I sit at work | ☐ | ☐ | ☐ | ☐ | ☐ |
| b. | recorded my sitting or standing at work in a written record | ☐ | ☐ | ☐ | ☐ | ☐ |
| c. | paid attention to specific things to help me stand at work (e.g., I have more energy in the morning so I stand during this time) | ☐ | ☐ | ☐ | ☐ | ☐ |
| d. | set short-term goals (daily or weekly) related to how often I stand up at work | ☐ | ☐ | ☐ | ☐ | ☐ |
| e. | broken down larger goals into smaller, more manageable goals (e.g. accumulate 40 minutes of standing in 4 x10minute bouts) | ☐ | ☐ | ☐ | ☐ | ☐ |
| f. | thought about my standing goals | ☐ | ☐ | ☐ | ☐ | ☐ |
| g. | reminded myself of the health benefits of standing at work (e.g., reduced risk of Type 2 diabetes, premature death) | ☐ | ☐ | ☐ | ☐ | ☐ |
| h. | scheduled specific times to stand up at work | ☐ | ☐ | ☐ | ☐ | ☐ |
| i. | paid attention to barriers which got in the way of my standing at work | ☐ | ☐ | ☐ | ☐ | ☐ |
| j. | planned ways to overcome barriers to my standing at work | ☐ | ☐ | ☐ | ☐ | ☐ |

**6. The next questions are about how often you used various strategies to help you stand up at work. If you haven't had a chance to use these strategies yet, please indicate 'Never' in the questions below. In the *past month* I have:**

| | Never | Rarely | Some-times | Often | Very Often |
|---|---|---|---|---|---|
| a. moved my workstation from sitting to standing or vice versa | ☐ | ☐ | ☐ | ☐ | ☐ |
| b. used my workstation tracker to record the days that I reach my standing goal at work | ☐ | ☐ | ☐ | ☐ | ☐ |
| c. used my workstation tracker to record my strategies for standing up and moving more at work | ☐ | ☐ | ☐ | ☐ | ☐ |
| d. used an on-line timer to time my periods of sitting or standing | ☐ | ☐ | ☐ | ☐ | ☐ |
| e. stood up when someone approached me at my workstation | ☐ | ☐ | ☐ | ☐ | ☐ |
| f. stood up when my phone rings | ☐ | ☐ | ☐ | ☐ | ☐ |
| g. walked to talk to a colleague (*iMail)* rather than sending them an eMail | ☐ | ☐ | ☐ | ☐ | ☐ |
| h. walked to the printer that is further away from my workstation | ☐ | ☐ | ☐ | ☐ | ☐ |
| i. walked to the bathroom that is further away from my workstation | ☐ | ☐ | ☐ | ☐ | ☐ |

**7. The next questions ask about your confidence in undertaking various activities. You may not have tried the activities listed - that is okay - but please still answer every question.**

**In the *past month*, if you were trying to reduce your sitting time at work, how confident would you have been that you could have:**

| | Not at all confident | Slightly confident | Somewhat confident | Confident | Very confident |
|---|---|---|---|---|---|
| a. stood up during meetings at work, even though no one else was | ☐ | ☐ | ☐ | ☐ | ☐ |
| b. stood up during meetings at work, even when supervisors were sitting down | ☐ | ☐ | ☐ | ☐ | ☐ |

| | | | | | |
|---|---|---|---|---|---|
| c. stood up at your desk at work, even though your colleagues were not | ☐ | ☐ | ☐ | ☐ | ☐ |
| d. stood up at your desk at work, even when you felt tired | ☐ | ☐ | ☐ | ☐ | ☐ |
| e. stood up at your desk at work, even if your footwear was uncomfortable | ☐ | ☐ | ☐ | ☐ | ☐ |
| f. stood up at your desk at work, even though you were really busy at work | ☐ | ☐ | ☐ | ☐ | ☐ |
| g. stood up at your desk at work, even when your tasks required looking at multiple papers | ☐ | ☐ | ☐ | ☐ | ☐ |
| h. stood up at your desk at work, even when your tasks required talking on the phone | ☐ | ☐ | ☐ | ☐ | ☐ |
| i. walk to talk to a colleague at work instead of emailing them, even though others didn't | ☐ | ☐ | ☐ | ☐ | ☐ |

*The above materials have been developed for use within the Stand Up Victoria study. Any future use of these materials must be referenced to this article.*
