# Supplementary material for: Reducing office workers’ sitting time: rationale and study design for the Stand Up Victoria cluster randomized trial
Source: BMC Public Health. 2013 Nov 9;13:1057. doi: 10.1186/1471-2458-13-1057 (PMC3828481; doi:10.1186/1471-2458-13-1057)
Supplement: Additional file 9: Figure S8 — Adverse Events. [file 1471-2458-13-1057-S9.pdf]

Adverse Events

1. Have you experienced any health problem(s) in the past 3 months which you believe is/are related to your participation in the study? *(please circle answer)*

No *(you have now completed this form)*

Yes *(please complete questions below)*

2. Please list health problem(s) below – one per line

(You may list up to three separate health problems below.)

1.
2.
3.

3. Was treatment sought from a health provider for this/these problems? *(please circle answer)*

No *(you have now completed this form)*

Yes *(please complete questions below)*

4. Please select type(s) of health providers and indicate number of visits:

Treatment type	Tick all that apply	Number of visits
General Practitioner (GP)	☐	
Massage therapist	☐	
Occupational therapist	☐	
Optometrist	☐	
Orthotist / Prosthetist	☐	
Podiatrist	☐	
Physiotherapist	☐	
Radiographer / Radiologist	☐	
Sonographer	☐	
Other *(please specify)*		

The above materials have been developed for use within the Stand Up Victoria study. Any future use of these materials must be referenced to this article.
